# Femtosecond Laser Produced Hydrophobic Hierarchical Structures on Additive Manufacturing Parts

**DOI:** 10.3390/nano8080601

**Published:** 2018-08-07

**Authors:** Lishi Jiao, Zhong Yang Chua, Seung Ki Moon, Jie Song, Guijun Bi, Hongyu Zheng

**Affiliations:** 1 Singapore Centre for 3D Printing, School of Mechanical and Aerospace, Nanyang Technological University, Singapore 639798, Singapore; jiao0011@e.ntu.edu.sg (L.J.); chuazy@ntu.edu.sg (Z.C.); 2 Singapore Institute of Manufacturing Technology, Singapore 637662, Singapore; songj@simtech.a-star.edu.sg (J.S.); gjbi@SIMTech.a-star.edu.sg (G.B); hyzheng@SIMTech.a-star.edu.sg (H.Z.); 3 School of Mechanical Engineering, Shandong University of Technology, Zibo 255000, China

**Keywords:** additive manufacturing, hydrophobic surface, nanostructures, laser processing

## Abstract

With the recent expansion of additive manufacturing (AM) in industries, there is an intense need to improve the surface quality of AM parts. A functional surface with extreme wettability would explore the application of AM in medical implants and microfluid. In this research, we propose to superimpose the femtosecond (fs) laser induced period surface structures (LIPSS) in the nanoscale onto AM part surfaces with the micro structures that are fabricated in the AM process. A hierarchical structure that has a similar morphology to a lotus leaf surface is obtained by combining the advantages of liquid assisting fs laser processing and AM. A water contact angle (WCA) of 150° is suggested so that a super hydrophobic surface is achieved. The scanning electron microscopy (SEM) images and X-ray photoelectron spectroscopy (XPS) analysis indicate that both hierarchical structures and higher carbon content in the laser processed area are responsible for the super hydrophobicity.

## 1. Introduction

Additive manufacturing (AM) is increasingly and making inroads in many industries which include automotive, aerospace, electronics, and biomedical areas [1]. There are many advantages of AM technologies over other manufacturing processes including the following: parts can be made easily on-demand for customization and personalization, special tooling is not required in part fabrication, the time cost of manufacturing and materials can be reduced significantly for individualized parts and small-quantity productions, novel components and structures of complex geometries and functional graded components can be fabricated without difficulties for some AM technologies, and the supply chain is compressed drastically [2].

However, research is still required to fully realize the potential of AM. The widespread adoption of AM is always challenged by the quality of the AM parts. In the past several years, most of the efforts focused on the internal quality such as porosity [3], mechanical properties [4], and dimensional accuracy [5]. Nevertheless, to achieve the real application of the AM components, we cannot neglect the importance of their external quality; surface quality such as wettability would affect the corrosion behaviors [6,7] and water–solid interaction performance of the AM fabricated device.

Traditional methods to produce hydrophobic surfaces include plasma treatment [8], chemical coating [9], and wet-chemical etching [10]. These methods are usually applied on a massive area that cannot be accurately defined onto a specific geometry unless using a mask with high expense. However, the durability of this hydrophobic surface is limited due to delamination of chemical coating.

Recently, intensive studies are focused on the interaction between laser energy and solid surfaces to create hierarchical structures on a solid to change the wettability properties [11,12,13]. The laser processing is a top to bottom, non-contact, and highly selective method that can be used to produce complex features. The femtosecond laser holds many advantages over other kinds of laser in material processing. Fs laser–material interaction is different from that of long pulse laser which induces a significant amount of heat and melting. In the case of the fs laser, the laser pulse duration (typically ~200 fs) is smaller than electron–phonon coupling time (in ps time scale) [14]. The laser energy can be deposited into a thin layer in a short time which is faster than lattice heating. The material removal process is confined in small volume enabling a precise machining process with minimal heat affected zone. Additionally, interaction between the laser pulse and plasma is avoided because the short laser pulse ends before the generation of plasma. This would increase the laser processing efficiency.

Fs laser energy with a small thermal effect can be tightly focused onto a tiny spot to create micro and/or nano structures that mimic hierarchical morphology from the natural world such as lotus leaves and shark skin, whose surface usually exhibits extreme non-wettability [15].

However, the laser treating from the above studies is mainly conducted on a flat or polished surface which is usually not applicable for AM parts. A high surface roughness cannot be avoided due to the nature of the AM process [16]. Additionally, the AM parts always have complex 3D features that are not possible to grind or polish using conventional methods.

In this study, we propose the implementation of a fs laser to directly fabricate nanostructures on the Ti6Al4V AM parts with pre-designed microstructures. A hierarchical structure with a super hydrophobic surface could be obtained by combining the advantages of the fs laser and AM process.

## 2. Experiment

**Figure 1:**
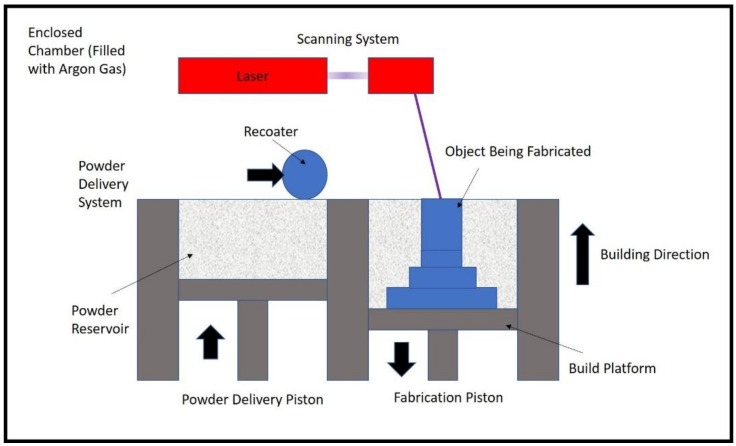
Schematic diagram of the selective laser melting (SLM) process.

One of the established metal AM processes is selective laser melting (SLM) which is capable of producing full-density, high-precision, and functional metal parts. SLM uses a powder deposition method consisting of a recoating mechanism to spread a powder layer onto a substrate plate and a powder reservoir. Once the powder is evenly distributed, SLM uses a high-power laser to trace the geometry of an individual layer of the slices from a 3D model on the surface of the powder bed. During the process, the powder particles are fused together, and solidification takes place. After a layer has been completed, the build platform is lowered down by the required layer thickness and the solidification process is continued to build up the finished part as shown in Figure 1.

To perform a set of experiments for surface properties modification, samples of 10 mm × 10 mm × 10 mm were fabricated by the SLM-250HL system (SLM solution GmbH, Lübeck, Germany) using the ytterbium laser (CW) source with a maximum power of 400 W. The SLM-250HL system offers a build volume of 250 × 250 × 250 mm³ and the laser beam is of 80 µm diameter. Ti6Al4V powders supplied by TLS Technik GmbH & Co (Bitterfeld-Wolfen, Germany) were used to manufacture all the test specimens. The size of the powders ranges from 20 to 63 µm and the chemical compositions (wt %) of the powder include: aluminum (6.46), vanadium (4.24), iron (0.17), oxygen (0.094), nitrogen (0.01), carbon (0.007), hydrogen (0.002), and titanium (balance). Table 1 shows the list of the SLM parameters used in this study.

The prepared AM samples were then processed by Quantronix Integra-C fs laser that emitted pulses of 130 fs with linearly polarized light at a central wavelength of approximately 795 nm. The nominal repetition rate is 1 kHz. The total pulse energy was attenuated by a rotating half wave. The mechanical shutter was controlled to release the desired laser on the substrate. The laser beam was focused and controlled by Scanlab galvanometer scanner. The average laser power after the lens was measured using a power meter. An area of 5 × 5 mm^2^ were processed in ethanol and air by using laser parameters listed in Table 2 on Ti6Al4V AM parts, each experiment was repeated three times.

The surface properties of the samples were analyzed using the Jeol JSM 5600-LV scanning electron microscope (SEM) and X-ray photoelectron spectroscopy (XPS) (ESCALAB 250Xi, Thermo Scientific, Paisley, UK). The surface water contact angle (WCA) measurement was conducted by applying the sessile drop method. A contact angle meter (Attension Theta Auto D) was used for the water contact angle measurement.

## 3. Results and Discussion

### 3.1. Partially Melted Powder

**Figure 2:**
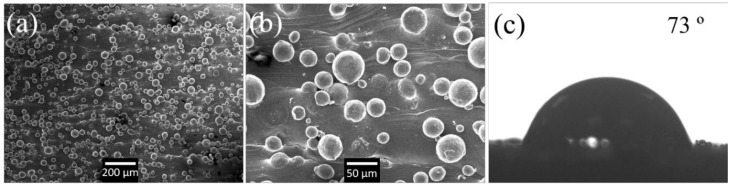
Scanning electron microscopy (SEM) image of the additive manufacturing (AM) surface covered with half-melt micro-particles (**a**) and (**b**); water droplet on the structures (**c**).

The partially melted micro-powder particles (PMP) are observed on the vertical walls as shown in Figure 2. In the SLM process, the powders in the center of laser track would be fully melted to create a melting pool. However, for high melting point metallic powders such as stainless steel [17] and Ti alloy [18], the powder right at the edge of laser track could be partially melted in the SLM process (powder bed based). When the laser beam moved away and the melting pool solidified, some parts of certain powder particles would be fused together with the solid part. The remaining part of this particle would be preserved as its original spherical shape. By choosing the scanning parameters in Table 1, the side wall can be covered with the partially melted particles. The existence of these PMPs is important because they constitute the micro structures that are considered as the first grade of the hierarchical surface.

After the manufacturing process, the samples were wire cut and removed from the substrate. The surface morphology of the samples was carefully analyzed using the SEM as shown in Figure 2a,b. The size of observed micro-sphere ranges from 20 to 60 µm that is similar with the raw powders. The water contact angle (WCA) of the sample that did not undergo laser processing was measured for reference purposes. This as-deposited SLM sample shows a hydrophilic surface (WCA 73°) as illustrated in Figure 2c.

**Figure 3:**
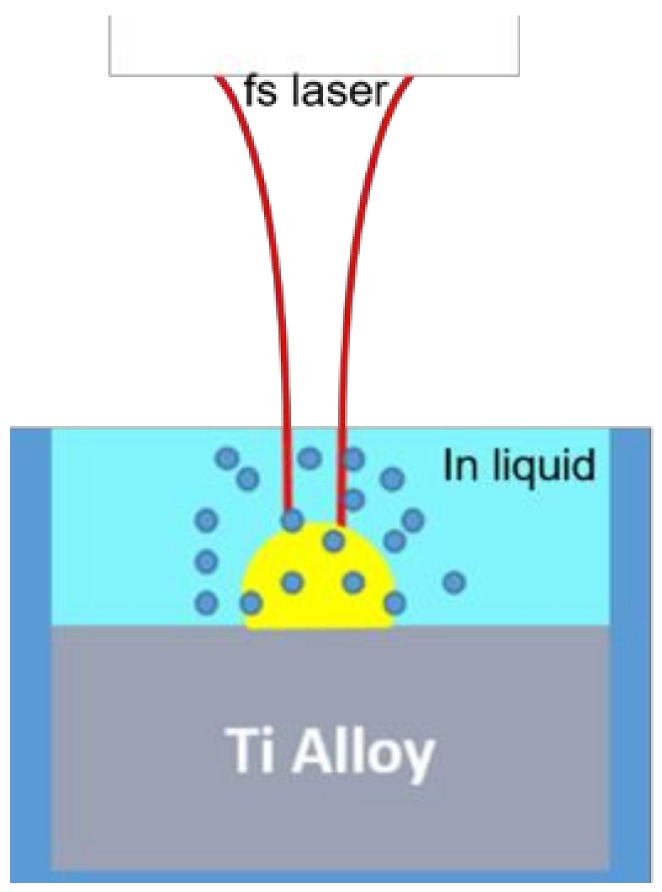
Setup of laser processing AM parts immersed in liquid.

As illustrated in Figure 3, the AM sample was immersed in the ethanol solution during the firing of the laser pulses. The thickness (assumed uniformly distributed here with relatively low surface tension) of liquid film is estimated to be 2 mm.

**Figure 4:**
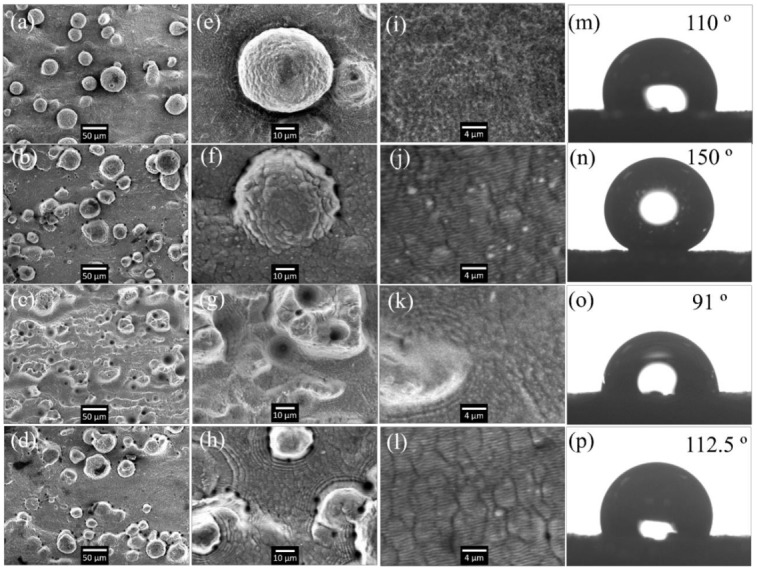
SEM images of the AM parts surface processed by fs laser in ethanol with scanning speed of 80 mm/s (**a**,**e**,**i**; first row); in ethanol with scanning speed of 40 mm/s (**b**,**f**,**j**; second row); in ethanol with scanning speed of 20 mm/s (**c**,**g**,**k**; third row); in air with scanning speed of 40 mm/s (**d**,**h**,**l**; last row); water droplet and water contact angle (WCA) on the corresponding structures (**m**–**p**).

The surface microstructures of the laser processed AM parts characterized by SEM are shown in Figure 4. The 1st, 2nd, 3rd rows show the profile of samples processed in ethanol liquid with a scanning speed of 80 mm/s, 40 mm/s, and 20 mm/s, respectively. To better illustrate the detailed features of the surface structures, the 2nd and 3rd columns show the SEM image captured at a higher magnification. As indicated in Figure 4a, the high speed laser scanning (80 mm/s) only has a minimal modification on the original surface. On the top view of the PMP, it shows a sphere shape with small distortion on the edge and no regular nano structures were found. When the scanning speed is decreased to 40 mm/s, the surface roughness is observed to expressly increase as shown in Figure 4b.

It is known that the long pulse laser or CW laser processing would cause re-melting and completely reformat the original surface structure. Ma et. al. [16] reported that the nanosecond (ns) fiber laser processed the AM made Ti alloys samples. In Ma et. al.’s study, the scanning of a ns laser with considerable thermal effect resulted in deep re-melting of the surface structures. The surface tension and gravity of the melting materials can make the liquid surface flat. When the melting surface re-solidified, the roughness of the sample is thereby reduced from 5 µm to 1 µm. The huge heat input from the ns laser also caused heat affect zone with thickness of 150 µm in which the material’s microstructures and metallurgical performance is modified.

However, fs laser processing with moderate fluence does small damage to the pre-design microstructures (i.e. the micro PMP still exists) due to the ultrashort pulse duration and minimal thermal effect. Figure 4f exhibits there is more laser induced distortion of the whole surface, while the PMP (microscale convex structures) can still be well preserved.

### 3.2. Laser Induced Periodical Surface Structures

Figure 4j is the further magnified image around the same area, which shows nano-ripples with a spatial wavelength of ~470 nm. These self-organized ripples are known as laser induced period surface structures (LIPSS) resulting from the interference of the incident laser induced surface scattered waves [19].

The nonuniform surface energy distribution from the interference leads to surface modification to form the final ripple status. The orientation of the LIPSS are perpendicular to the incident laser polarization direction and their spatial wavelength is usually found to be close to or smaller than the incident laser’s wavelength [20]. The period of ripple produced here in ethanol is around 470 nm, which is about 40% less than the incident laser wavelength (795 nm). The laser processing environment of ethanol has a refractive index of 1.36 compared to that of air of 1.0. According to the physical model in the literature [19], the spatial wavelength can be significantly smaller than incident laser wavelength when the refractive index of processing medium is increased. The observation of 470 nm period is consistent with the previous literature [21] that applied a 775 nm fs laser on silicon in ethanol.

The SEMs in the third row of Figure 4 show the sample that underwent laser processing at a slower scanning speed of 20 mm/s. At this speed, most of the PMPs are damaged and micro-holes with a diameter of 5–20 µm are formed. Excessive energy input leads to a drastic material removal process that damages the original structures and no LIPSS can be found as well.

The last row of the Figure 4 is the sample processed in air using the exact same laser parameters as second row. Without ethanol’s influence, the LIPSS’s wavelength is measured as around 520 nm that is also smaller than the laser wavelength. This observation is consistent with the recent literature [22] that produced LIPSS with a period of 520 nm on pure polished titanium by a 790 nm fs laser. Theoretically, the modulus of the surface plasmon wave vector has a strong connection with the surface morphology. [23] It is well accepted that the LIPSS’s period would be decreased with increasing the real part of the surface plasmons’ refractive index. Previous literature reported that the initial laser pulses can create some random nanostructures including nanorods, nanocones, and nanospheres. [24] These nanostructures would contribute to the increase of the real part of the surface plasmons’ refractive index. In most of the cases, the generation of LIPSS requires multiple laser pulses. With applying more numbers of pulses, more nanostructures are thereby produced, allowing a smaller LIPSS period.

With selected laser processing parameters (listed in Table 2), large area typical LIPSSs are imposed on the PMP dominated AM surface. The surface covered by hierarchical structures including micro features (20–60 µm) and nano features (470 nm) is obtained.

### 3.3. Wettability

The wettability of the laser processed surface is characterized using static WCA as shown in Figure 4m–p. All the WCA measurement shown in Figure 4 were performed 10 days after laser processing. The WCAs reveal significant variation among the different surface structures. Comparing to the as-deposited surface with WCA of 73° in Figure 2c, all the samples in Figure 4 show a larger WCA resulting from the increase of surface roughness induced by fs laser processing. This observation is consistent with Cassie-Baxter’s model which assumes the air can be trapped between the solid structures and water. A composite interface would be formed and the WCA increases with surface roughness [25].

Importantly, Figure 4n shows the WCA is increased to 150° on the hierarchical structures that are fabricated in ethanol. The LIPSS has a strong influence on WCA. It is known that [26] the nanostructures (LIPSS) can pin the liquid-air interface and thus prevent liquid from filling the valleys between microstructures (PMP). It is important for the LIPSS to support nanodroplets that would not easily condensate in the gaps between microstructures.

The samples (as shown in Figure 4m,o) without LIPSS exhibit smaller WCAs than the ones with LIPSS. These results concluded that the combination of the micro and nano structures results in highest WCA.

It is worth noting that there is obvious LIPSS existing on the surface processed in air, however, the WCA of this sample is much small than its counterpart that is processed in ethanol. It was observed that the LIPSS fabricated in liquid is 10% finer than that in air. The smaller nano feature size and correspondingly large surface area may enhance the hydrophobicity of the sample processed in ethanol.

### 3.4. Chemical Composition

In addition, it is known that the surface chemistry has significant influence on the wettability. To make a comparison for the chemical composition of the sample produced in different environment, chemical bonds of all of the surface are analyzed by XPS (10 days after the laser processing). Table 3 exhibits the relative atomic percentage of elements for the surface of samples that underwent non-processing (NP), laser processing in air (PA), and processing in liquid (PL). It is found that carbon, oxygen, and titanium are the main elements for all the three samples. NP sample shows a high carbon content of 35.75%. This XPS detected C may have two sources: the first source is the contamination from the coolant solvent during the wire cutting which is an essential process to separate the AM sample from the substrate; the second source can be the hydrocarbon from air because the sample is stored in an air environment for three months after AM fabrication. After laser processing, both the PA and PL samples show a decrease in the C content. It can be explained by the laser induced local temperature rise that can easily burn the surface carbon. Additionally, the high pressure generated by plasma expansion [27] during laser processing could blow away the contamination adhering to the surface. The surface carbon content is usually considered to have a positive effect on the hydrophobicity. However, the NP sample with the highest C content exhibits the smallest WCA which is mainly due to their less hydrophobic surface morphology.

It is observed that the O content form TiO_2_ that is not hydrophobic [28] is higher in the PA sample than the PL sample. For the PA sample, Oxygen is mainly induced by the dramatic oxidation during the laser processing that can cause a thick oxidation layer. For the cases of the PL sample that are processed in liquids, the ethanol liquid would insulate the air from the ablation area thus avoiding the fast and massive oxidation of titanium at high temperature.

**Figure 5:**
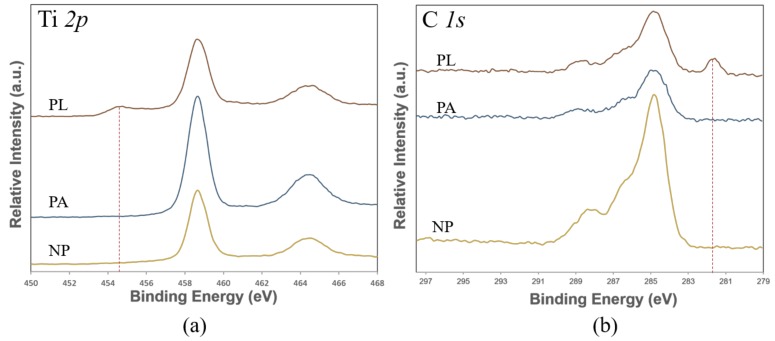
XPS spectra of Ti 2p (**a**) and C *1s* (**b**) for sample underwent non-processing (NP), laser processing in air (PA) and in liquid (PL).

The O content from the PL sample is due to the slow oxidation during storage in air. As shown in Figure 5a, the peak of metallic Ti (454.57 eV) still can be observed in XPS spectra even at the 10th day after laser processing.

On the other hand, the fs laser fluence is sufficient to result in the photo-dissociative ionization of ethanol to break the covalent bonds (C–C and C–O) [29]. This can increase the possibility of chemical reaction between Ti– and C– at the solid-liquid interface. This process can be validated by the appearance of a C–Ti peak (281.64 eV) in the XPS spectra of PL sample. It can have a contribution to the higher C content in the PL sample (23.94%) than that in the PA sample (13.95%). The deconvoluted N 1s peak at 400.4 eV corresponds to organically bound nitrogen (C–N). This nitrogen possibly comes from the nitrogen containing carbon contaminants (proteins or oil in air) that might be brought in during storage or transfer.

The observed carbon may be related to the nonpolar functional groups including methylgroup-CH_3_ and/or graphitic carbon that are hydrophobic [30]. These functional groups possibly are responsible for the high hydrophobicity of the PL sample.

**Figure 6:**
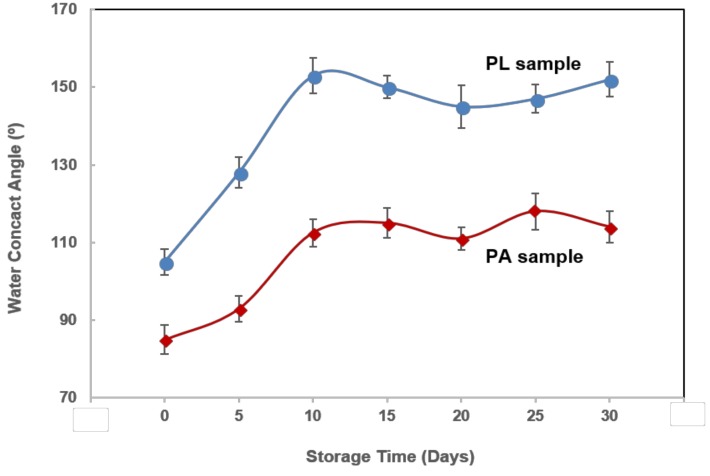
WCA development of PL and PA samples stored in air.

During the WCA testing, it is found that the WCA increases with storage time and reaches a relatively steady state after around 10 days as shown in Figure 6. This phenomenon is usually attributed to the increased carbon content along with storage time. For the case of the PL sample, it is believed a certain portion of the carbon comes from the Ti-C_2_H_5_OH reaction in the laser processing. The other portion is due to the accumulation of carbon [31] possibly sourced from the airborne hydrocarbon contaminations [32] during the storage. The laser creates nanostructures with higher surface energy can increase the surface adsorption activities [32] and may help to capture the airborne hydrocarbon. This also could be the main reason for the carbon accumulation in the PA sample. After 10 days, the WCAs for both PA and PL sample show small fluctuations that may come from the slow change of the surface chemistry. It is known that there are still some chemical reactions and physical adsorption activities happening on the surface sample that was stored in air. [28] The oxidation of pure titanium produces TiO_2_ which is hydrophilic. However, continuous accumulation of carbon would result in hydrophobic features. With increasing storage time, the competing of several phenomena could cause fluctuations of WCA.

## 4. Conclusions

In this study, a facile approach to produce functional graded surface on AM parts was proposed. The implementation of liquid assisted fs laser processing created periodical surface ripples with a spatial wavelength of 470 nm on the Ti6Al4V AM sample that was covered by predesigned PMP with diameter from 20 to 60 µm. The combination of LIPSS in nanoscale and PMP in microscale enabled the hierarchical structures that had a similar morphology to the surface of a lotus leaf. The WCA of 150° suggested that a super hydrophobic surface was obtained when the LIPSS was fabricated in ethanol at a laser scanning speed of 40 mm/s. It is believed that both the hierarchical structures and higher surface carbon content contribute to the super hydrophobicity. These results can pave a way for AM parts to obtain water repelling and self-cleaning properties that would explore the application area of AM.

## Figures and Tables

**Table 1:** Processing parameters used in the SLM of TI6AL4V samples.

Parameters	Unit	Values
Laser Power	W	95
Layer Thickness	mm	0.050
Scan Speed	mm/s	125
Hatch space	mm	0.11

**Table 2:** Femtosecond (fs) laser processing parameters.

Parameters	Unit	Values
Scan Speed	mm/s	20, 40, 80
Hatch density	mm	0.02
Laser spot size	µm	120
Laser intensity	W/cm^2^	4.9 × 10^13^

**Table 3:** Relative atomic percentage of element obtained from X-ray photoelectron spectroscopy (XPS).

Sample Name	Relative Atomic Percentage of Detected Elements					
	Al 2p	C 1s	N 1s	O 1s	Ti 2p	V 2p
Non-processed (NP)	3.86	36.75	4.41	42.96	12.01	0.30
Processed in air (PA)	5.58	14.95	1.23	54.89	23.35	0.21
Processed in liquid (PL)	4.09	23.94	1.64	50.49	19.84	0.46
